# Interaction of ZEB and Histone Deacetylase with the PLDLS-binding cleft region of monomeric C-terminal Binding Protein 2

**DOI:** 10.1186/1471-2199-10-89

**Published:** 2009-09-15

**Authors:** Ling-Jun Zhao, M Kuppuswamy, S Vijayalingam, G Chinnadurai

**Affiliations:** 1 Institute for Molecular Virology, Saint Louis University Health Sciences Center, St. Louis, Missouri 63104, USA

## Abstract

**Background:**

Proteins of the C-terminal binding protein (CtBP) family, CtBP1 and CtBP2 are closely related transcriptional regulators that are coded by two different gene loci in the vertebrate genomes. They perform redundant and unique functions during animal development. CtBP proteins mediate their transcriptional function through interaction with various DNA-binding repressors that contain PLDLS-like motifs and chromatin modifying enzymes, such as class I histone deacetylases (HDAC) that do not contain such motifs. The N-terminal region of CtBP1/2 forms a hydrophobic cleft and is involved in interaction with both PLDLS-containing factors and non-PLDLS factors. CtBP proteins function as dimers to mediate transcriptional repression and dimerization is modulated by specific binding to NAD/NADH.

**Results:**

In this study, we have investigated the role of dimerization of CtBP2 in recruitment of PLDLS-motif cofactors and non-PLDLS cofactors. Our results indicate that mutations in CtBP2 that interfere with dimerization abolish CtBP2 interaction with most cellular factors, except the PLDLS-motif factor zinc-finger E-box binding homeobox (ZEB) and the non-PLDLS factor HDAC2. Unlike most PLDLS-containing CtBP-binding proteins, ZEB contains three PLDLS-like motifs and all three contribute to the interaction with the CtBP2 monomer. Despite the ability to interact with ZEB and HDAC, the CtBP2 monomer fails to mediate ZEB-dependent transcriptional repression. The lack of repression activity of the CtBP2 monomer is correlated with the competition between ZEB and HDAC for interaction with the CtBP2 monomer.

**Conclusion:**

These results suggest a competition between the canonical PLDLS-motif factors such as E1A and non-PLDLS factor HDAC for interaction with CtBP. They also indicate that the affinity for the CtBP monomer may be determined by the number as well as amino acid sequence compositions of the PLDLS-like motifs. Our results are consistent with a model that the CtBP2 dimer may interact with a PLDLS-containing repressor through one monomer and recruit HDAC and other chromatin modifying enzymes through the second monomer in the CtBP2 dimer.

## Background

The adenovirus E1A C-terminal binding protein (CtBP) was identified as a cellular protein that specifically binds with the PLDLS-motif in E1A and regulates the transforming activity of E1A [1-3]. Subsequent studies have shown that CtBP is an evolutionarily conserved transcriptional co-repressor that is utilized by a variety of vertebrate and invertebrate transcriptional repressors [4]. CtBP1/2 regulate expression of genes that control cell differentiation [5], proliferation [6-8] and apoptosis [9], with critical consequences on oncogenesis [10].

The CtBP2 locus codes for three splice variants - CtBP2-L (referred to here as CtBP2), CtBP2-S and Ribeye. CtBP2-L is localized in the nucleus while CtBP2-S and Ribeye that lack a unique N-terminal region (NTR) present in CtBP2-L are cytosolic [11-14]. The CtBP2 NTR is acetylated by p300 [11] and this modification is linked to the subcellular localization of CtBP2. CtBP1 which lacks such a domain is believed to localize in the nucleus by alternate mechanisms including heterodimerization with CtBP2-L and interaction with nuclear transcription factors [12,13]. Structural studies have revealed that CtBP1 is a dimer and the structure is substantially similar to that of 2-hydroxy acid dehydrogenases [15-17]. CtBP2 also has a similar structure (Pelka et al., Protein Data Bank, ID 2OME). The N-terminal region and C-terminal region of CtBP form a hydrophobic cleft to interact with cofactors that contain motifs similar to the canonical CtBP-binding motif, PLDLS present in adenovirus E1A. In addition to the core PLDLS motif, adjoining sequences may also influence the affinity of interaction [18].

*In vitro *studies have shown that dimerization of CtBP1 is required for interaction with E1A [19]. NAD(H)-mediated dimerization has been reported to enhance the transcriptional repression activity of CtBP [20,21]. However, it is unclear whether the CtBP monomer can interact with PLDLS-containing factors *in vivo *and mediate transcriptional repression. Although CtBP1 mutants deficient in dimerization are defective in transcriptional repression, it is difficult to ascribe this lack of activity to co-factor recruitment since such mutants of CtBP1 are deficient in nuclear localization [22].

Transcriptional repression by CtBP proteins appears to be dependent on simultaneous interaction of CtBP with both a DNA-binding repressor and a chromatin modifying enzymes, such as HDAC [23]. Importantly, the cleft region of CtBP1 appears to be involved in interaction of both PLDLS-motif factors and non-PLDLS factors [22]. While the PLDLS-dependent interaction between CtBP and repressors has been well-characterized, the interaction between CtBP and HDAC remains unclear. Since CtBPs are dimers, the presence of two different cleft regions in the dimer would complicate the analysis of interaction of these factors with the cleft region. Here, we have used a monomeric mutant of CtBP2 that is capable of nuclear localization under the control of the cognate nuclear localization signal [12,13,24]. We have discovered that the CtBP2 monomer can interact with a major CtBP-dependent repressor ZEB as well as HDAC and that the interaction of the two factors with the CtBP2 monomer was mutually exclusive. These results have led us to formulate a model by which the CtBP dimer could assemble repression complexes by simultaneous interaction with a PLDLS-motif factor and a non-PLDLS factor.

## Methods

### Cell culture, transfection, immunofluorescent staining, and luciferase assays

HeLa, 293, MCF7 and CtBP1/2 double knock-out MEF90 cells were cultured in DMEM supplemented with 10% fetal bovine serum and penicillin/streptomycin. Transfection reagent jetPEI was used for transient transfection for luciferase assay, and Lipofectamine 2000 was used for transfection for co-immunoprecipitation and immunofluorescence analyses as previously described [11]. For luciferase assays, cells were transfected in 24-well plates in duplicates. Average luciferase activity was plotted, with the control luciferase activity normalized to 1.0. Average deviation was plotted as the error bar. For co-immunoprecipitation analysis, cells were transfected in 100 mm dishes, and cell lysates were immunoprecipitated with the Flag antibody beads (Sigma). When the HBH tag (His6-biotinylation-His6) was used, transfected cells were treated with 5 μM biotin for the duration of transfection, and immunoprecipitation was performed with the streptavidin-agarose beads (Pierce). Immunofluorescence analysis was performed with the Cy3-conjugated Flag antibody (Sigma).

### DSS cross-linking

Cells in 12-well plates were incubated with 5 mM Dissuccinimidyl suberate (DSS) (Pierce) in the culture medium for 30 min. After washing with PBS, cells were lysed with 200 μl of 2× SDS sample loading buffer and examined by western blots as described in the text.

### Plasmid constructs

The ZEB [GenBank:NM_030751] region coding for aa #680-780 containing the PLDLS-like motifs was PCR-amplified with primer N309 **(**ACATGCGGATCC*CTCGAG*CCTTTGAAGATGACTAACTCCCCA) and N297 (AGCTACGAATTCTTAGTCCTTTTGTGGCTCCTTTTTTGCG), digested with BamHI/EcoRI, and cloned into the vector pGFP-E1A [25] to generate pGFP-ZEB101. Mutants of ZEB101 were cloned similarly by using an overlapping PCR approach [11]. For expression of GST-ZEB101, ZEB101 region was transferred from pGFP-ZEB101 to a pGST vector at the XhoI/EcoRI sites. Mutants of ZEB101 were transferred to the GST vector by the same approach. RR-CtBP2 and GG-CtBP2 mutants were generated based on the pFH-CtBP2 construct [11] using the Quick-Change kit (Stratagene). The primers for RR-CtBP2 (R147L/R169L) were #7065 (CCATCTGCCACATCCTCAACCTGTACGCCAGGAACACGTGGCTGTACCAGGCACTGCGG) and #7067 (GGCACGCGGGTTCAGAGCGTGGAGCAGATCCTTGAGGTGGCCTCGGGAGCGGCCCGCATC). The primer for GG-CtBP2 (G189A/G192A) was #7063 (GGCCCGCATCCGTGGGGAGACGCTGGGCCTCATTGGCTTTGCTCGCACGGCTCAGGCGGTTGCAGTTCGAGCCAAGGCC). Other CtBP constructs were described earlier [11,25].

### GST pull-out assay

GST fusion constructs were induced with 1 mM IPTG for 2 h for expression of GST fusion proteins. Lysates containing the desired GST fusion proteins were loaded onto glutathione-agarose beads in the binding buffer containing 25 mM Tris-HCl (pH7.5), 0.25 M NaCl, 0.5% Triton X-100, 0.5 mM DTT, 0.1 mM PMSF, and the protease inhibitor cocktail (Roche). Subsequently, purified H6-CtBP2 and H6-RR-CtBP2 (~0.1 μg each) were incubated with the pre-charged glutathione-agarose beads in 200 μl of the binding buffer for 1 h at 4°C. After washing, bound CtBP2 proteins were examined by Western blot with the CtBP2 monoclonal antibody and the GST fusion proteins examined by Coomassie blue staining.

## Results

### Analysis of CtBP2 dimerization

**Figure 1:**
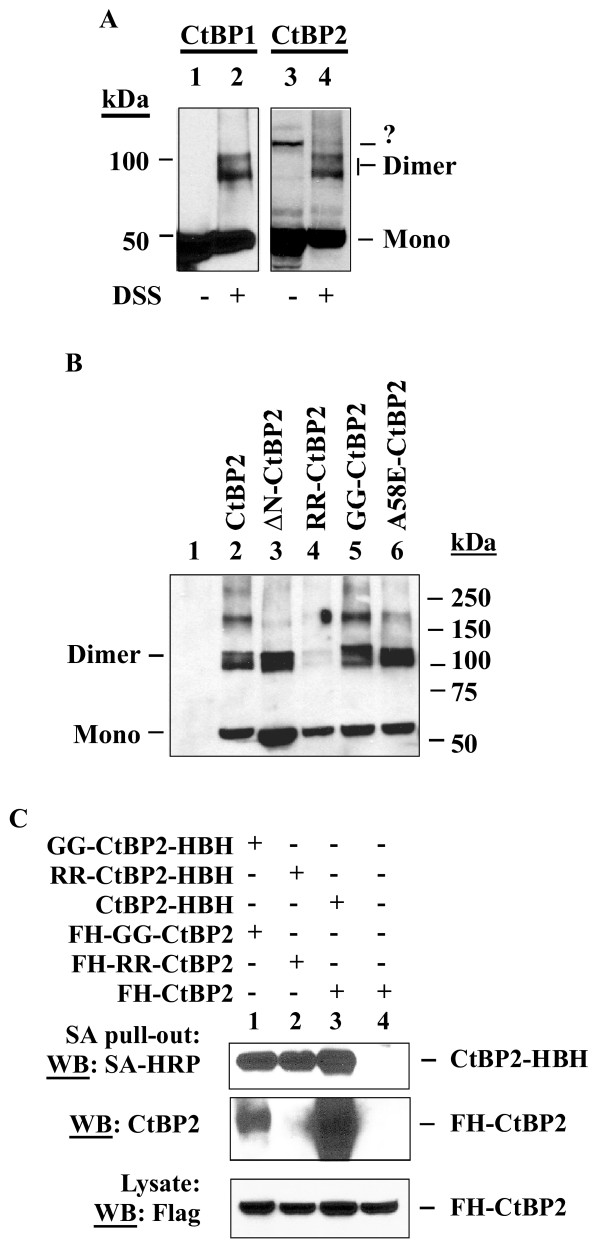
**Analysis of dimerization of CtBP2 and its mutants**. *A*, Dimerization of endogenous CtBP proteins. HeLa cells were treated with DSS and Western blots performed with CtBP1 and CtBP2 antibodies (Pharmingen). *B*, Dimerization of transiently expressed CtBP proteins. Various Flag-HA-tagged CtBP constructs were transfected into HeLa cells. One day after transfection, cells were treated with DSS and cell lysates were examined by Western blot analysis using the Flag antibody. *C*, Dimerization defect of RR-CtBP2 and GG-CtBP2. CtBP2-HBH, RR-CtBP2-HBH, or GG-CtBP2-HBH was co-transfected with the Flag-HA-tagged version in indicated combinations. Biotin (5 μM) was added to cells after addition of DNA precipitates. After immunoprecipitation with the streptavidin (SA) agarose beads, bound proteins were analyzed by western blot with a CtBP2 antibody to detect FH-CtBP2, or with the streptavidin-HRP conjugate to detect CtBP2-HBH proteins.

First, we examined the role of various structural elements of CtBP2 in dimerization using the cross-linking approach. To validate this approach, we carried out western-blot analysis of HeLa cells exposed to the cross-linking agent DSS (Fig. 1A). Higher molecular weight bands that corresponded to dimers of CtBP1 and CtBP2 were readily detected while no such dimers were detected in untreated cells. We noticed a doublet in the position of the endogenous CtBP1 and CtBP2 dimers. Although the exact reasons for the formation of such doublets are unknown, it may be possible that they are due to heterodimerization of CtBP1 and CtBP2, and/or cross-linking of CtBP to other cellular proteins. In addition, the high molecular weight band in lane 3 of Fig. 1A in the absence of DSS could be a non-specific band or an alternative high order form of CtBP2 that is resistant to denaturing SDS-PAGE conditions.

To examine the roles of the various CtBP2 domains in CtBP2 dimerization, Flag-HA-tagged CtBP2 and mutants were transfected into HeLa cells. After DSS cross-linking, cell lysates were examined by western blot analysis using the Flag antibody. As shown (Fig. 1B), deletion of the unique CtBP2 N-terminal region (NTR) did not affect the extent of dimerization, but severely reduced the ability of CtBP2 to form higher order forms (compare lanes 2 with 3). Mutations R147L/R169L (mutant RR) corresponding to the critical residues in the predicted dimerization interface of CtBP1 [15,22] severely reduced dimer formation as well as other higher order forms of CtBP2 (lane 4). Despite the reported effects of NAD(H)-binding on CtBP1 dimerization, a mutation in the NAD(H)-binding motif of CtBP2 (G189A/G192A, mutant GG) did not affect CtBP2 dimerization by this analysis (lane 5). Similarly, a mutation within the PLDLS-binding cleft (A58E) that severely reduces interaction with various PLDLS-motif factors also did not affect dimerization.

To determine whether the RR-CtBP2 and GG-CtBP2 mutants were capable of forming dimers under normal (without cross-linking) conditions, HeLa cells were co-transfected with Flag-HA tagged CtBP2 or various mutants, and their corresponding versions with a C-terminal HBH tag. The HBH tag contains a biotinylation signal sequence sandwiched between two (His)6 tags [26]. Cell lysates were adsorbed with the streptavidin-agarose affinity beads, and the precipitated proteins examined by western blots as indicated. As shown in Fig. 1C, wt CtBP2-HBH dimerized efficiently with the wt FH-CtBP2 (lane 3), whereas the streptavidin-agarose beads did not pull out FH-CtBP2 alone (lane 4). The RR-CtBP2-HBH mutant did not dimerize with FH-RR-CtBP2 (lane 2). Interestingly, by this approach GG-CtBP2-HBH was found to form a dimer with FH-GG-CtBP2 (lane 1), but at a greatly reduced level compared to the wt CtBP2 (lane 3). Thus, the RR-CtBP2 mutant may have a reduced level of dimerization which is only detectible under cross-linking conditions. In contrast, the NAD(H)-binding mutant GG-CtBP2 remains capable of forming a dimer, which appears to be less stable under co-immunoprecipitation conditions and could be stabilized by cross-linking with DSS (Fig. 1B).

### Co-factor recruitment by monomeric CtBP2

**Figure 2:**
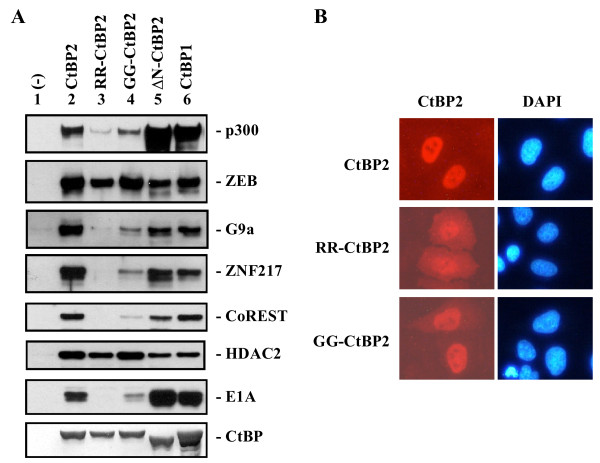
**Interaction of co-factors with CtBP2 mutants**. *A*, Western blot analysis of CtBP2 protein complex. Flag-HA tagged CtBP proteins were transiently expressed in 293 cells. Cell lysates were immunoprecipitated with the Flag antibody and western blots performed with the antibodies indicated. *B*, Subcellular localization of CtBP2 wt and mutants. HeLa cells were transfected with indicated plasmids and immunofluorescence analysis performed with Cy3-Flag antibody.

To examine the effects of CtBP2 dimerization and NAD(H)-binding on interaction with cellular and viral co-factors, Flag-HA-tagged CtBP2 and the RR-CtBP2 and GG-CtBP2 mutants were transfected into 293 cells, which express endogenous E1A, and CtBP2 complexes were immunoprecipitated from the cell lysates with the Flag antibody. Western blot analysis of the CtBP2 complexes showed that the dimerization mutant, RR-CtBP2 (Fig. 2A, lane 3), and the mutant in the NAD(H)-binding motif, GG-CtBP2 (lane 4), bound to ZEB and HDAC2 well. However, binding of mutant RR-CtBP2 to other factors was defective. In contrast, GG-CtBP2 (lane 4) bound to other co-factors much less efficiently than the wt CtBP2 but much more efficiently than RR-CtBP2 (lane 3). These results suggest that dimerization and NAD(H)-binding are both critical for CtBP2 interaction with co-factors. The reduced efficiency of GG-CtBP2 binding to most co-factors appears to be correlated with the reduced stability of the GG-CtBP2 dimer (Fig. 1).

To compare co-factor binding by CtBP2 and CtBP1, FH-CtBP1 was also included in the analysis (Fig. 2A, lane 6). As shown, CtBP1 bound to p300 and E1A more efficiently than CtBP2, whereas CtBP2 bound to HDAC2 more efficiently than CtBP1. Interestingly, deletion of the CtBP2 N-terminal domain (lane 5) rendered CtBP2 to behave similarly to CtBP1. Thus, the unique N-terminal region (NTR) of CtBP2 appears to regulate CtBP2 interaction with co-factors.

Previous results have shown that the unique CtBP2 NTR allows CtBP2 to be localized completely in the nucleus while CtBP1 lacking the NTR is localized in both the cytoplasm and the nucleus [11]. To examine the role of dimerization for CtBP2 nuclear localization, Flag-HA-tagged CtBP2, RR-CtBP2, and GG-CtBP2 were transfected into HeLa cells, and their subcellular localization detected with the Flag antibody. As shown in Fig. 2B, while the wt CtBP2 and GG-CtBP2 were predominantly localized in the nucleus, RR-CtBP2 had significant cytoplasmic localization. Previous analysis of a CtBP1 dimerization mutant suggested that it was localized exclusively in the cytoplasm [22]. Thus, it appears that while the dimerization mutations reduced the efficiency of CtBP2 nuclear localization, the CtBP2 NTR remains functional in targeting the RR-CtBP2 mutant to the nucleus.

### Dimerization and NAD(H)-binding in CtBP2 repression of E-cadherin (E-cad) promoter

**Figure 3:**
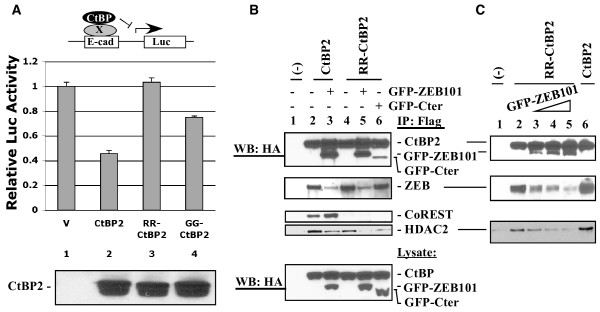
**Transcriptional repression by CtBP2 and mutants and competition between HDAC and ZEB for RR-CtBP2 binding**. *A*, Repression of E-cad promoter by CtBP2 and mutants. CtBP1/2 double knock-out cell line MEF90 cells were co-transfected with pE-cad-Luc, phRL-tk (for internal control), and various CtBP2 constructs. Dual luciferase assay was performed as described [11]. Lysates were examined for CtBP expression by western blot with the CtBP2 antibody (lower panel). *B*, Interaction of wt CtBP2 and RR-CtBP2 with endogenous ZEB and HDAC2 in the presence of GFP-ZEB101. HeLa cells were co-transfected with CtBP2 and GFP-ZEB101. Cell lysates were immunoprecipitated with the Flag antibody beads and precipitated proteins examined by western blots as indicated. Both CtBP proteins and GFP fusion proteins carry an HA tag and are recognized by the HA antibody. *C*, Competition between GFP-ZEB101 and HDAC2 for binding to RR-CtBP2. HeLa cells were co-transfected with CtBP2 and increasing amounts of GFP-ZEB101. Co-IP and western blot analyses were performed as in B.

Transcriptional repression by CtBP1 and CtBP2 requires CtBP interaction with both DNA-targeting transcription repressors, such as ZEB [5,27-29], and enzymes involved in chromatin modification/remodeling, such as HDAC1/2 [23]. Since both RR-CtBP2 and GG-CtBP2 interacted with ZEB well (Fig. 2A), we examined the ability of these CtBP2 mutants to repress the E-cad promoter. Although several different repressors have been implicated in repression of the E-cad promoter, ZEB appears to play a dominant role [5,11,23]. CtBP2 wt or its mutants were co-transfected with the E-cad-Luc reporter into the CtBP1/2 double knock-out cell line, MEF90. Luciferase assay showed that GG-CtBP2 retained significant repression activity, while RR-CtBP2 had no repression activity (Fig. 3A). Thus, the ability of RR-CtBP2 to interact with ZEB (Fig. 2A) appears to be insufficient for repression of the E-cad promoter.

### Binding of HDAC and ZEB to the CtBP2 monomer is mutually exclusive

We previously reported that CtBP2 repression of E-cad promoter was dependent on HDACs [11]. Our results in Fig. 2A indicated efficient interaction of HDAC2 and ZEB with RR-CtBP2 in co-immunoprecipitation studies. Since RR-CtBP2 interacts with ZEB but does not repress the E-cad promoter (Fig. 3A), it is possible that RR-CtBP2 interaction with ZEB and with HDAC is mutually exclusive. To examine this possibility, the ZEB region (aa #680-780), which contains three candidate PLDLS-like motifs, was cloned as a fusion construct, GFP-ZEB101. Flag-HA-tagged CtBP2 or RR-CtBP2 was transfected into HeLa cells alone or together with GFP-ZEB101. Lysates were immunoprecipitated with the Flag antibody and the precipitated proteins analyzed by western blots (Fig. 3B). As shown, both CtBP2 and RR-CtBP2 bound to GFP-ZEB101 as expected (lanes 3 and 5, first panel from top). Control co-transfection of RR-CtBP2 with GFP-Cter, which expresses a GFP fusion protein containing the E1A C-terminal region encompassing the PLDLS motif [30] showed that RR-CtBP2 bound to GFP-Cter much less efficiently (lane 6, first panel), consistent with the observation that RR-CtBP2 was defective in binding to cellular endogenous E1A in 293 cells (Fig. 2A). Interaction of CtBP2 or RR-CtBP2 with GFP-ZEB101 resulted in inhibition of interaction with the cellular endogenous ZEB (lanes 3 and 5, second panel), but did not affect CtBP2 interaction with CoREST (third panel). RR-CtBP2 binding to HDAC2 was severely competed by GFP-ZEB101 (lane 5, fourth panel), and was competed to a lesser extent by GFP-Cter (lane 6), consistent with the lower efficiency of RR-CtBP2 binding to GFP-Cter. CtBP2 wt binding to HDAC2 was competed to some extent by GFP-ZEB101 (lane 3). However, CtBP2 retained a higher level of HDAC2 binding than RR-CtBP2 in the presence of GFP-ZEB101 (compare lanes 3 and 5).

To substantiate the conclusion that ZEB and HDAC2 bound to RR-CtBP2 competitively, RR-CtBP2 was co-transfected with increasing amounts of GFP-ZEB101 into HeLa cells and co-immunoprecipitation analysis was again performed (Fig. 3C). As shown, with increasing amounts of GFP-ZEB101, binding of endogenous ZEB and HDAC2 to RR-CtBP2 was progressively reduced. Thus, the competition between ZEB and HDAC2 binding to RR-CtBP2 may be partly accountable for the defective transcriptional repression of the E-cad promoter by RR-CtBP2 (Fig. 3A).

### Interaction of ZEB with the CtBP2 monomer requires multiple PLDLS-like motifs

**Figure 4:**
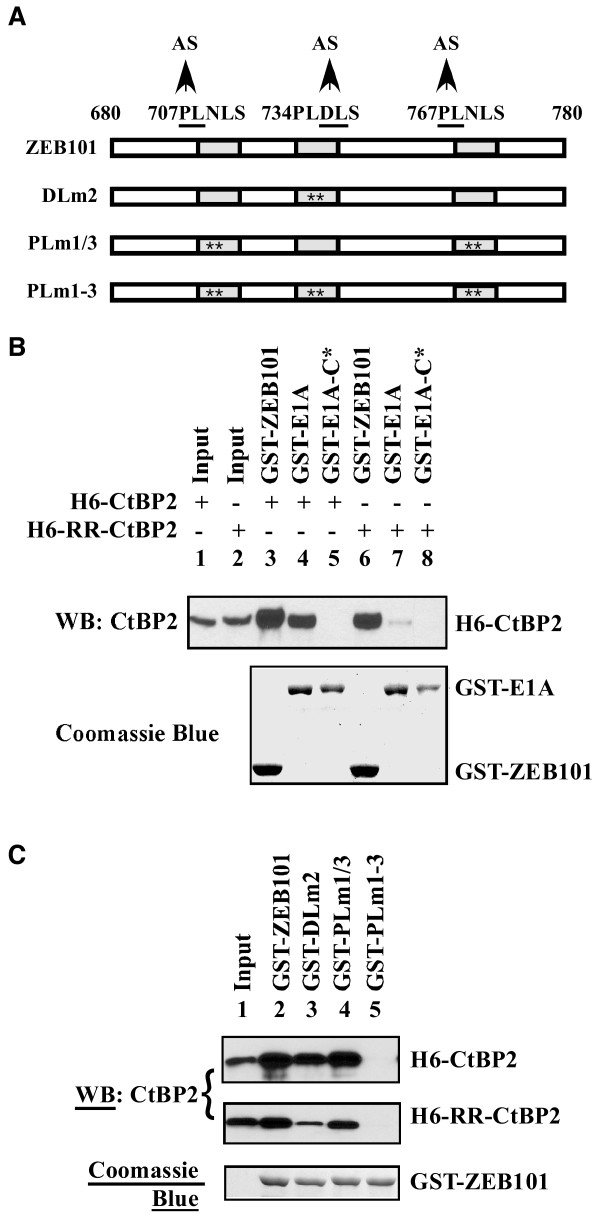
**Roles of PLDLS-like motifs of ZEB in interaction with CtBP2 and RR-CtBP2**. *A*, Diagram of constructs. Numbers denote the amino acid residue number in the full-length ZEB1. *B*, Interaction of CtBP2 and RR-CtBP2 with GST-ZEB101 and GST-E1A. GST-ZEB101, GST-E1A, and GST-E1A-C* (with a DL→AS mutation in PLDLS motif) were charged to glutathione beads and then incubated with purified H6-CtBP2 or H6-RR-CtBP2. Bound CtBP2 was examined by western blot with the CtBP2 antibody and GST-fusion proteins visualized by Coomassie blue. Input (lanes 1 and 2) represents 5% of input proteins. *C*, Interaction of CtBP2 and RR-CtBP2 with mutants of GST-ZEB101. Conditions were the same as in B, except that Input (lane 1) represent 10% of input proteins. Bottom panel: Coomassie blue-stained GST-fusion proteins for the binding assays involving H6-CtBP2 (top panel). GST-fusion proteins for the binding of H6-RR-CtBP2 were the same (not shown).

The near normal interaction between RR-CtBP2 and ZEB was in sharp contrast to the defective interaction between RR-CtBP2 and E1A (Fig. 2A and Fig. 3B). Within the CtBP-interaction domain of ZEB, there are three PLDLS-like motifs (Fig. 4A) - a central PLDLS motif, and a PLNLS motif on both sides. The middle motif is identical to the canonical CtBP-binding motif of E1A [31]. To directly examine the roles of the individual PLDLS-like motifs in interaction with wt CtBP2 and RR-CtBP2, ZEB101 region was expressed as a GST-ZEB101 fusion protein. Mutants of GST-ZEB101 were constructed as shown in Fig. 4A, to incorporate mutations in the PLDLS-like motifs. The DLm2 mutant contains DL→AS mutations in the central PLDLS motif, the PLm1/3 mutant contains PL→AS mutations in the two PLNLS motifs, and the PLm1-3 mutant contains mutations in all three motifs.

GST-ZEB101 and GST-E1A were first used for *in vitro *pull-out assays to examine their binding to H6-tagged wt CtBP2 and RR-CtBP2. As shown in Fig. 4B, while GST-ZEB101 bound to both wt CtBP2 and RR-CtBP2 efficiently (compare lanes 3 and 6), GST-E1A only bound to wt CtBP2 efficiently (lane 4) but not to RR-CtBP2 (lane 7). Neither wt CtBP2 nor RR-CtBP2 interacted with the GFP-E1A-C* mutant (lanes 5 and 8), which carried a DL→AS mutation within the PLDLS motif. These results are consistent with the observation *in vivo *that ZEB bound to RR-CtBP2 at near normal levels, whereas E1A did not bind to RR-CtBP2 (Fig. 2A). Thus, the CtBP2 monomer appears to preferentially interact with ZEB both *in vivo *and *in vitro*.

GST-ZEB101 mutants with mutations in various PLDLS-like motifs were also analyzed for their interaction with H6-CtBP2 and H6-RR-CtBP2 (Fig. 4C). For this analysis, comparable amounts of H6-CtBP2 and H6-RR-CtBP2 were used (as indicated in lane 1 of top and middle panels). In all lanes bound H6-CtBP2 and H6-RR-CtBP2 were examined on the same western blot, and were arranged as the top (for H6-CtBP2) and middle (for H6-RR-CtBP2) panels for comparison. As shown, despite a small reduction in interaction between H6-CtBP2 and ZEB101-DLm2 (lane 3, top panel), H6-CtBP2 (top panel) interacted efficiently with the wt ZEB101 (lane 2), as well as the two mutants (lanes 3 and 4). In contrast, H6-RR-CtBP2 (middle panel) bound to GST-ZEB101 and mutants less efficiently than H6-CtBP2 (top panel). In particular, H6-RR-CtBP2 bound to GST-ZEB101 (middle panel, lane 2) much better than the ZEB101-DLm2 mutant (middle panel, lane 3). The ZEB101-PLm1/3 mutant (lane 4) bound to RR-CtBP2 only slightly less efficiently than the wt ZEB101 (lane 2). As expected, mutation of all three PLDLS-like motifs rendered ZEB101 incapable of interaction with both the wt CtBP2 (top panel, lane 5) and the RR-CtBP2 mutant (middle panel, lane 5). These results suggested that all three PLDLS-like motifs in ZEB101 may contribute to the interaction with RR-CtBP2. Among the three motifs the central PLDLS motif appears to be most important.

### Nuclear targeting of GFP-ZEB by CtBP2 monomeric mutant

**Figure 5:**
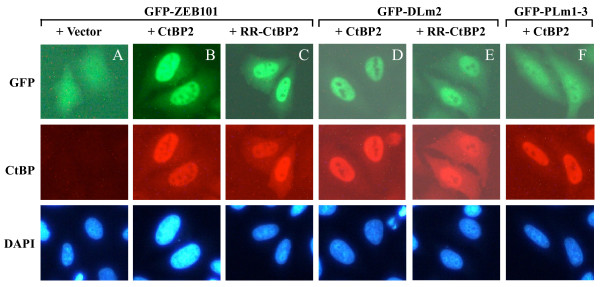
**Enhancement of nuclear localization of GFP-ZEB101 by RR-CtBP2**. GFP-ZEB fusion constructs were co-transfected with Flag-HA-tagged CtBP2 or RR-CtBP2. One day after transfection, cells were fixed and stained with Cy3-conjugated Flag antibody to visualize CtBP2 and RR-CtBP2. Localization of GFP-ZEB fusion proteins was indicated by the fluorescence of GFP.

We previously reported that CtBP2 promotes nuclear localization of a cytoplasmically localized E1A mutant [11]. GFP-ZEB101 was localized throughout the cell in a diffused pattern (Fig. 5A, top panel), consistent with the fact that the ZEB sequence does not contain a nuclear localization signal. Coexpression of GFP-ZEB101 and CtBP2 wt resulted in nuclear localization of GFP-ZEB101 (Fig. 5B, top panel). Interestingly, co-expression of GFP-ZEB101 with RR-CtBP2 also resulted in nuclear localization of GFP-ZEB101 (Fig. 5C, top panel), despite the reduced efficiency of nuclear localization for RR-CtBP2 compared to wt CtBP2 (Fig. 2B). It should be noted that RR-CtBP2 nuclear localization seems to be slightly enhanced by co-transfection with GFP-ZEB101 (compare Fig. 5B and 5C with Fig. 2B). It might be possible that interaction of RR-CtBP2 with a PLDLS-containing partner helps stabilize the conformation of RR-CtBP2 to enhance the nuclear localization of RR-CtBP2. In contrast, the subcellular localization of GFP-ZEB-PLm1-3 was only marginally changed by co-expression of wt CtBP2 (Fig. 5F), consistent with the observation that interaction of wt CtBP2 and RR-CtBP2 with GST-ZEB-PLm1-3 was defective (Fig. 4C). Although RR-CtBP2 interacted with GST-ZEB-DLm2 *in vitro *much more weakly than wt CtBP2 (Fig. 4C), both wt CtBP2 and RR-CtBP2 enhanced nuclear localization of GFP-ZEB-DLm2 (Fig. 5D and 5E) suggesting that even a weaker interaction between RR-CtBP2 and GFP-ZEB-DLm2 may be sufficient for enhanced nuclear localization of GFP-ZEB-DLm2. Thus, both the CtBP2 dimer and CtBP2 monomer appear to be capable of promoting the nuclear localization of their interaction partners.

### Transcriptional repression by RR-CtBP2 when tethered to the promoter by Gal4 fusion

**Figure 6:**
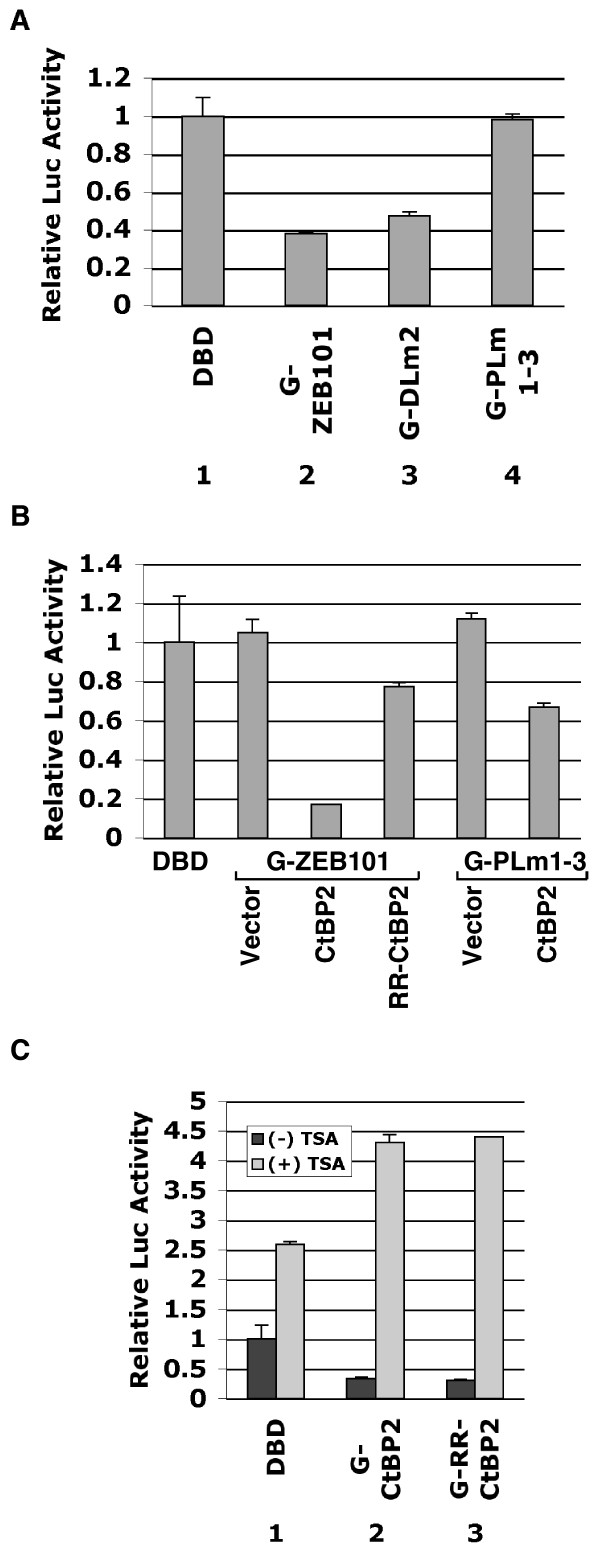
**HDAC-dependent transcriptional repression by Gal4-RR-CtBP2**. *A*, Transcriptional repression by Gal4-ZEB101. Gal4 fusion constructs were co-transfected into MCF7 cells with pG5-Luc and phRL-tk. Dual luciferase assay was performed as in Fig. 3A. DBD: Gal4 DNA binding domain. *B*, Failure of RR-CtBP2 to repress transcription through Gal4-ZEB101. CtBP1/2 double knockout cells were co-transfected with pG5-Luc, phRL-tk, Gal4-ZEB101 and CtBP2 or RR-CtBP2. Dual luciferase assay was performed as in A. *C*, HDAC-dependent transcriptional repression by Gal4-CtBP2 and Gal4-RR-CtBP2. MEF90 cells were co-transfected with pG5-Luc, phRL-tk, and Gal4-CtBP2 or Gal4-RR-CtBP2. HDAC inhibitor TSA was added at 0.2 ng/ml during transfection. Dual luciferase assay was carried out as in A.

To examine the functional correlation between CtBP2 interaction with GFP-ZEB101 and CtBP2 transcriptional repression, ZEB101 was expressed as a Gal4-ZEB101 fusion protein. Co-transfection of Gal4-ZEB101 with the luciferase reporter, G5-MLP-Luc, into MCF7 cells resulted in transcriptional repression (Fig. 6A). Similarly, Gal4-ZEB-DLm2 was also active in repression, consistent with the ability of GST-ZEB-DLm2 to interact with both the CtBP2 monomer and CtBP2 dimer (Fig. 4). In contrast, Gal4-ZEB-PLm1-3 did not repress transcription, possibly due to its defective interaction with CtBP.

When Gal4-ZEB101 was co-transfected with G5-MLP-Luc into MEF90 cells with CtBP1/2 double knock-out, it did not repress transcription (Fig. 6B), consistent with the CtBP deficiency in MEF90 cells. However, co-transfection of Gal4-ZEB101 with CtBP2 resulted in transcriptional repression. In contrast, co-transfection of Gal4-ZEB101 with RR-CtBP2 had a much lower transcriptional repression activity. These results are consistent with the observation that RR-CtBP2 binding to ZEB and HDAC2 is mutually exclusive (Fig. 3B and 3C). When Gal4-ZEB-PLm1-3 was co-transfected with CtBP2, a low level of transcriptional repression was observed. However, the level of activity was much less compared to that mediated by Gal4-ZEB101. The transcriptional repression activity mediated by Gal4-PLm1-3 could be due to a low level of *in vivo *interaction between Gal4-PLm1-3 and CtBP2. Thus, the ability of Gal4-ZEB101 to repress transcription appears to be correlated with interaction of endogenous CtBP2 through the PLDLS-like motifs in Gal4-ZEB101.

The ability of CtBP2 and RR-CtBP2 to directly repress transcription was also examined by the Gal4-tethering assay (Fig. 6C). As shown, both Gal4-CtBP2 and Gal4-RR-CtBP2 were active in repression of transcription. Thus, once tethered to the promoter by Gal4, the CtBP2 monomer is capable of transcriptional repression. Importantly, when the HDAC inhibitor TSA was included during transfection, transcriptional repression by both Gal4-CtBP2 and Gal4-RR-CtBP2 was abolished. Thus, Gal4-tethered transcriptional repression by CtBP2 and RR-CtBP2 appeared to be mostly mediated by HDAC.

## Discussion

The transcriptional repression function of CtBP corepressors appears to depend on simultaneous recruitment of DNA-binding repressors and chromatin modifying enzymes by CtBP. CtBP interaction with DNA binding repressors is usually mediated through PLDLS-like motifs in the repressors [4,32]. In some cases, it also involves an auxiliary motif known as the RRT motif present in repressors such as Znf217 [33]. In contrast to our understanding of the mode of interaction of PLDLS-motif and PLDLS/RRT motif factors, the mode of interaction of chromatin modifying enzymatic constituents of the CtBP corepressor complex remains less well defined. A detailed mutagenesis study of CtBP1 has indicated that the PLDLS-binding cleft of CtBP1 plays a critical role in recruiting these factors [22]. Certain amino acid substitution mutations within the cleft region of CtBP1 [22] and CtBP2 [25] affect interaction with both PLDLS-motif factors and HDAC1/2. In an earlier study, we have attempted to investigate interaction of CtBP cofactors with a monomeric mutant of CtBP1 [22]. Since CtBP1 monomeric mutants were excluded from the nucleus, we used a heterologous (SV40) nuclear localization signal to target such mutants to the nucleus. The interaction of most cofactors with such chimeric mutant was substantially increased suggesting that the observed interaction may not reflect the normal pattern of interaction possibly due to localization of the mutant in non-relevant nuclear sub-compartment. Thus, our present analysis of cofactor interaction with the monomeric CtBP2 targeted to the nucleus *via *the cognate targeting mechanism appears to reflect the normal pattern of interaction.

We employed two different CtBP2 mutants, one with mutations in residues corresponding to residues of CtBP1 implicated in dimerization (RR-CtBP2) and the other in the conserved NAD(H)-binding motif (GG-CtBP2). Since no direct structural data is available for these mutants, potential unintended effects of these mutations on CtBP2 structure may not be excluded. Interestingly, both RR-CtBP2 and GG-CtBP2 bound to ZEB and HDAC2 to near normal levels (Fig. 2A). In contrast, GG-CtBP2 binding to other CtBP2 co-factors was greatly reduced, and RR-CtBP2 binding to these co-factors was mostly defective. These observations suggest that CtBP2 interaction with certain cofactors may be less dependent on dimerization or NAD(H)-binding, and raise the possibility that CtBP2 monomer may have unique functions. It should be noted that by DSS cross-linking analysis, RR-CtBP2 still retained a small degree of dimerization (Fig. 1B). The possibility that the low level of dimerization is required for RR-CtBP2 to interact with ZEB and HDAC2 could not be excluded. Examination of subcellular localization of CtBP2 and its mutants revealed that while GG-CtBP2 and wt CtBP2 assumed a predominant nuclear localization pattern, RR-CtBP2 had significant cytoplasmic localization. Thus, although NAD(H)-binding does not appear to be critical for CtBP2 nuclear localization (Fig. 2B), it significantly affects CtBP2 interaction with co-factors other than ZEB and HDAC2. The reduced efficiency of RR-CtBP2 nuclear localization may be partially accountable for the reduced interaction of RR-CtBP2 with ZEB (Fig. 2A).

The observations with the mutant of CtBP2 in the NAD(H)-binding motif, GG-CtBP2, revealed important clues to transcriptional repression by CtBP2. The dimerization of the GG-CtBP2 mutant was normal as judged by DSS cross-linking (Fig. 1B). However, by co-immunoprecipitation analysis the GG-CtBP2 mutant dimerized at a greatly reduced efficiency compared to the wt CtBP2 (Fig. 1C). These results seem to be consistent with the hypothesis that NAD(H)-binding induces conformational changes in CtBP to stabilize CtBP dimerization [21]. In CtBP1/2 double knock-out MEF90 cells, retrovirally-expressed GG-CtBP2 had similar activity as the wild type CtBP2 in E-cad promoter localization and E-cad transcriptional repression, whereas RR-CtBP2 expressed in this fashion was unstable and could not be analyzed (data not shown). These results are consistent with the finding that GG-CtBP2 binding to ZEB and HDAC2 was nearly normal (Fig. 2A), and GG-CtBP2 remains capable of transcriptional repression in transient reporter assays (Fig. 3A). Although E-cad transcriptional repression by CtBP2 does not appear to require NAD(H)-binding by CtBP2, it is possible that under certain physiological conditions NAD(H)-binding by CtBP2 becomes important for the transcriptional repression activity of CtBP2. Importantly, since GG-CtBP2 binding to co-factors other than ZEB and HDAC2 was severely reduced, it is expected that CtBP2 regulation of other potential candidate target genes is more dependent on NAD(H) binding than repression of E-cad.

Two important results have emanated from our present study with the monomeric CtBP2 - 1) CtBP2 co-factors have varying degrees of requirements for CtBP2 dimerization and NAD(H)-binding, and 2) the interaction of HDAC and PLDLS-motif factors with CtBP2 monomer might be mutually exclusive. The interaction between CtBP2 monomer and ZEB appears to be dependent on three PLDLS-like motifs. Mutational analysis of these motifs in ZEB suggest that the central PLDLS motif is most critical, while the two flanking PLNLS motifs appear to play auxiliary roles (Fig. 4). It is important to note that the PLDLS motif in E1A is identical to the central PLDLS motif of ZEB, and yet the GFP-E1A-Cter fusion protein interacted with the CtBP2 monomer much more weakly than GFP-ZEB101 (Fig. 3B), suggesting that auxiliary motifs may contribute to the unique binding activity of ZEB. Our results on ZEB binding with CtBP2 monomer agrees well with a previous report on ZEB binding with CtBP1 and repression assays, where all three sites were found to be required for efficient CtBP-binding and full repression activity [28]. Thus, the unique arrangement of the three PLDLS-like motifs in ZEB appears to contribute to the ability of ZEB to interact with the CtBP2 monomer. It seems possible that the two PLNLS motifs in ZEB may function as low affinity nucleation sites and the central PLDLS motif may function as the high affinity site for stable interaction with CtBP2.

It is possible that presence of two or more binding motifs among the CtBP binding factors may be more prevalent than previously recognized. For example, the presence of a PLNLS motif and the RRT motif in Znf217 facilitates interaction with the CtBP dimer [33]. The possibility that a similar mode of interaction may be used by Riz, Znf516 [33] and Wiz [22,34] has been pointed out. Our present study shows that the PLDLS and PLNLS motifs of ZEB are functionally redundant for interaction with the CtBP2 dimer. Two different divergent PLDLS-related motifs of EBV EBNA3B have been reported to synergize for binding with CtBP [35]. More recently a second binding site has been identified in adenovirus E1A, in addition to the PLDLS motif [36]. Presence of divergent and functionally redundant CtBP-binding motifs in transcription factors may regulate transcription by context-dependent recruitment of CtBP and chromatin modifying enzymes.

Our current analysis of cofactor interaction with the CtBP2 monomer has revealed new insights into the mechanism of the HDAC-dependent repression of the E-cad promoter by CtBP2. ZEB and HDAC2 appeared to compete for interaction with the CtBP2 monomer since co-expression of the CtBP2 monomer with GFP-ZEB101 severely hindered HDAC2 interaction with RR-CtBP2 (Fig. 3B and 3C). HDAC2 interaction with RR-CtBP2 was progressively reduced by increasing amounts of GFP-ZEB101 (Fig. 3C). This may account for the lack of repression of the E-cad promoter by the CtBP2 monomer (Fig. 3A). A CtBP2 monomer anchored to the E-cad promoter through interaction with ZEB may not be able to recruit repressive enzymes such as HDAC2 to the promoter. However, anchoring of the CtBP2 monomer onto a promoter through Gal4 fusion rendered the CtBP2 monomer capable of transcriptional repression (Fig. 6C), consistent with the conclusion that after Gal4 anchoring of RR-CtBP2 to the promoter, the CtBP2 monomer remains capable of recruiting HDAC. Although Gal4 functions as a dimer, it is expected that the Gal4-RR-CtBP2 "quasi" dimer is structurally and functionally different from the natural CtBP2 dimer, and the ability of Gal4-RR-CtBP2 to repress transcription resides in the ability of RR-CtBP2 to recruit HDAC.

**Figure 7:**
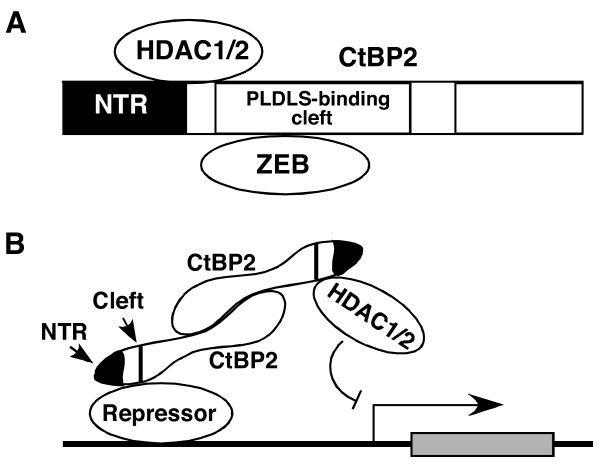
**Model for HDAC-dependent transcriptional repression by CtBP2**. *A*, Interaction of HDAC1/2 and ZEB with CtBP2 monomer. Based our on results we suggest that HDAC1/2 interact with CtBP2 sequences that encompass the hydrophobic cleft (PLDLS-binding) region and additional regions such as the NTR, while ZEB and other PLDLS-motif containing transcription factors interact exclusively with the cleft region. Our results suggest that interaction of the CtBP2 monomer with HDAC1/2 and the PLDLS-motif factors is mutually exclusive. *B*, Transcriptional repression by CtBP2. We propose that recruitment of CtBP2 with the DNA-bound PLDLS-motif containing repressor *via *one monomer of the CtBP2 dimer might facilitate recruitment of HDAC1/2 *via *the second CtBP2 monomer.

Our results are consistent with the hypothesis that strong interaction of PLDLS-motif factors such ZEB with the CtBP2 monomer may preclude interaction of the CtBP2 monomer with HDAC (Fig. 7A). These results also lend support to the model of transcriptional repression by CtBP2 (Fig. 7B), in which CtBP2 normally interacts with a DNA-bound repressor through one CtBP2 monomer, and recruits a DNA modifying enzyme, such as HDAC through the other CtBP2 monomer. In this context, it remains possible that interaction of one CtBP2 monomer with a repressor, such as ZEB, may help facilitate recruitment of HDAC through the other CtBP2 monomer.

## Conclusion

1) CtBP2 monomer retains ability to interact with ZEB but this interaction does not result in transcriptional repression.

2) CtBP2 monomer interaction with ZEB involves three PLDLS-like motifs, each of which contributes to interaction to various extents.

3) A mutant in the NAD(H)-binding motif of CtBP2 seems to interact with ZEB normally and repress E-cad transcription normally.

4) CtBP2 monomer interaction with ZEB and HDAC is mutually exclusive.

## Abbreviations

CtBP: C-terminal binding protein; ZEB: zinc-finger E-box binding homeobox; HDAC: histone deacetylase; NTR: N-terminal region; HBH: tag containing (His)6-biotinylation signal-(His)6; TSA: trichostatin A; DSS: dissuccinimidyl suberate.

## Authors' contributions

GC and L-JZ designed and wrote the paper; MK constructed the pFH-RR-CtBP2 and pFH-GG-CtBP2 clones and performed preliminary dimerization and luciferase assays; SV performed preliminary fluorescence analysis for the CtBP2 wt and mutants; L-JZ performed all experiments shown in the figures.
